# Subcutaneous Fat Obesity in a High Body Mass Index Donor Is Not a Contraindication to Living Donor Hepatectomy

**DOI:** 10.1155/2023/9540002

**Published:** 2023-07-29

**Authors:** Hirak Pahari, Amey Sonavane, Amruth Raj, Anup Kumar Agrawal, Ambreen Sawant, Deepak Kumar Gupta, Amit Gharat, Vikram Raut

**Affiliations:** ^1^Department of Liver Transplant and HPB Surgery, Medicover Hospitals, Navi Mumbai, Maharashtra, India; ^2^Department of Gastroenterology and Hepatology, Medicover Hospitals, Navi Mumbai, Maharashtra, India; ^3^Department of Radiology, Medicover Hospitals, Navi Mumbai, Maharashtra, India; ^4^Department of Liver Transplant Anaesthesia, Medicover Hospitals, Navi Mumbai, Maharashtra, India

## Abstract

**Background:**

Living donor liver transplantation (LDLT) has revolutionized the field of transplantation without compromising donor safety. Donor safety is of paramount concern to the transplant team. BMI >35 kg/m^2^ is mostly considered a contraindication to liver donation. Here, we present a successful right donor hepatectomy from a donor with a BMI of 36.5 kg/m^2^. *Case Summary.* A 39-year-old wife donated her right lobe of liver to her 43-year-old husband with nonalcoholic steatohepatitis-related chronic liver disease (CLD). His indications were refractory ascites, hepatic encephalopathy, acute kidney injury, recurrent elbow and urine infections leading to cachexia. She was initially rejected due to a high BMI but failed to lose weight over the next 2 months, and the need for a transplant in her husband was imminent. With no other potential living donors, we decided to proceed with donor evaluation as she had no other comorbidity. We were surprised to find normal liver function tests and a good liver attenuation index (LAI) of +16 on a computed tomography (CT) scan. Magnetic resonance (MR) imaging revealed a fat fraction of 3%. Volumetry confirmed a remnant of 37.9% and a potential graft-to-recipient weight ratio of 1.23. V/S ratio on CT scan (visceral fat area/subcutaneous fat area at L4-level) was <0.4 confirming subcutaneous fat obesity. Both surgeries were uneventful and both donor and recipient recovered well except recipient re-exploration on postoperative day (POD)-1 due to surgical bleeding. The donor was discharged on POD-6 and recipient was discharged on POD-15. At 3 weeks of follow-up, the donor's wound is clean and well-healed, and she is already back to doing her daily life activities without any pain with normal laboratory parameters.

**Conclusion:**

Subcutaneous fat obesity should not be considered as a contraindication to liver donation even with a BMI >35 kg/m^2^. A small percentage of healthy individuals will not have visceral fat obesity and may not have steatotic livers. The CT scan and MR fat fraction estimation can confirm the findings. Biopsy may be avoided if MR fat estimation is <10% in obese donors. Intraoperative visualization in these donors remains the gold standard to decide the need for biopsy. Living donor hepatectomy may be safely performed in a select group of high BMI patients (>35 kg/m^2^) with pure subcutaneous fat obesity in the absence of other suitable living donors.

## 1. Introduction

Living donor liver transplantation (LDLT) has revolutionized the field of transplantation since the 1990s, leading to a vast increase in the number of transplants worldwide without compromising donor safety. The donor is usually a healthy individual with minimal to no comorbidity, and donor safety is of paramount concern to the transplant team. The usual limits of donor acceptance in terms of age, body mass index (BMI), liver remnant, fat content of the liver (measured through liver attenuation index, LAI, and so on), and other parameters are well established [1, 2].

Most centers would accept donors with a BMI <30 kg/m^2^ while a BMI of 30–35 kg/m^2^ would still be evaluated for liver fat estimation [3, 4]. BMI >35 kg/m^2^ is mostly considered a contraindication to liver donation [1, 4]. High-BMI potential donors are frequently asked to lose weight prior to their evaluation as a living liver donor [5]. Liver fat is usually estimated by various methods, the most common ones being CT evaluation of the liver attenuation index (LAI) and magnetic resonance (MR) spectroscopy [1, 5]. A biopsy is often done to confirm the findings in high-BMI donors before proceeding with living liver donation [1, 5]. Herein, we present a case where the donor had a high BMI (>35 kg/m^2^) who underwent an uneventful right donor hepatectomy without the need for a liver biopsy.

## 2. Case Presentation

The patient is a 43-year-old male patient with nonalcoholic steatohepatitis (NASH)-related chronic liver disease (CLD) since 3.5 years. His weight prior to transplant was around 68 kgs, and his dry weight (after paracentesis) was 63 kgs. His predominant symptoms were refractory ascites with frequent large volume paracentesis which was gradually increasing in frequency, and one episode of hepatic encephalopathy. His Na-MELD (sodium-model for end-stage liver disease) score was 15, and he was on the waiting list for deceased donor liver transplantation since the last 2 years (blood group B positive). He was found to have developed a grade-2 near-complete portal vein thrombosis in the last 4 months, extending almost up to the splenomesenteric confluence. His low hemoglobin required him to have repeated transfusions every 2 months since the last 1 year. He also had left elbow swelling which had persistent infection with methicillin-resistant *Staphylococcus aureus* that was not responding to treatment. He had undergone multiple orthopedic interventions with no benefit.

At this time, he was counseled for living donor liver transplantation (LDLT) in September 2022, but he had no suitable donor. Apart from his 39-year-old wife, who had a matching blood group (O positive) but a high BMI of 36.5 kg/m^2^ (weight 81 kgs and height 149 cms), he did not have any potential liver donors. As per our protocol, we advised her for a regimen of diet and exercise and to return in 2 months for reassessment. During these 2 months, the patient got admitted 3 times with repeated elbow infection and swelling. Major orthopedic surgery could not be performed and the elbow was deformed in a position of flexion and medial rotation. He was also getting recurrent *Klebsiella* infection in his urine, and his creatinine increased to 1.88 mg/dl. He was getting more and more cachexic, and the need for a transplant was becoming more imminent.

**Figure 1:**
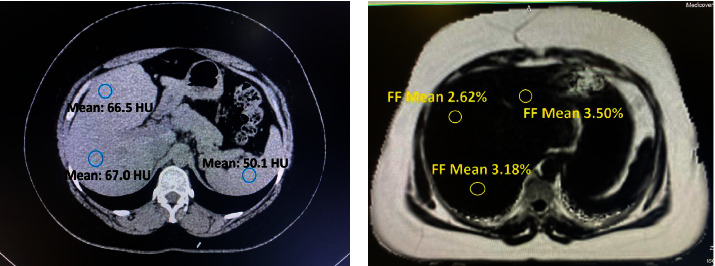
(a) LAI +16 on plain CT scan. (b) MR fat fraction 2-3% in both lobes (LAI = liver attenuation index).

**Figure 2:**
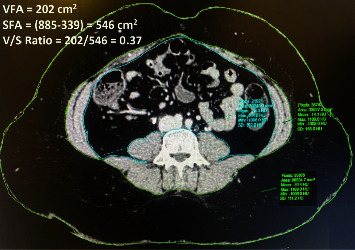
Visceral fat to subcutaneous fat (*V*/*S*) ratio <0.4 indicating subcutaneous obesity and not visceral obesity [6]. VFA: visceral fat area; SFA: subcutaneous fat area; *V*/*S*: VFA/SFA ratio taken at L4-level.

His wife returned to us after 2 months with no change in weight (81 kgs). Even with such a high BMI, we decided to evaluate her for donation. She had no significant medical or surgical history. To our surprise, her liver function tests, glycosylated hemoglobin (HbA1c), thyroid function, and fasting lipid profile were normal. The computed tomography (CT) estimation calculated liver attenuation of 66.4 Hounsfield units (HU) (right lobe 66.1 and left lobe 66.7) and a mean splenic attenuation of 49.6 HU, giving us an LAI of +16.5 and +17.1 for the right and left hepatic lobes, respectively (Figure 1(a)). We routinely perform magnetic resonance cholangiopancreatography (MRCP) in all donors, and we also ran MR fat estimation on her which revealed a fat content of approximately 3% in both the right and left liver lobes (Figure 1(b)). The V/S ratio, which is a ratio of visceral fat area to the subcutaneous fat, was 0.37 (<0.4 refers to subcutaneous obesity as compared to >0.4 in visceral obesity) [6] (Figure 2). Volumetric analysis was then performed which is outlined in Table 1 (Table 1). We opted for right-lobe living donor hepatectomy.

**Figure 3:**
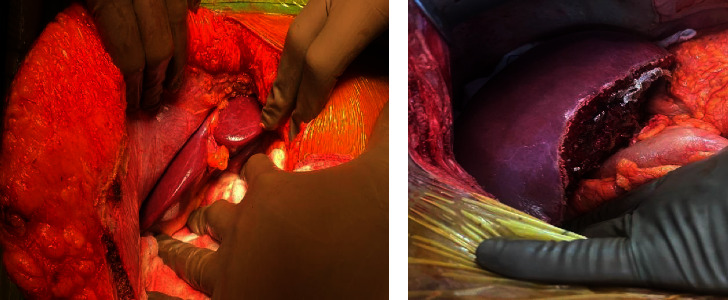
(a) The difference between visceral fat and abdominal wall fat in donor, indicating subcutaneous fat obesity. (b) After reperfusion in recipient, showing a healthy soft pink liver.

After completing the work-up of both patient and donor, we proceeded with LDLT on 13^th^ February, 2023. In the operating room, the donor liver looked healthy and nonsteatotic (Figure 3(a)). We did not proceed with a biopsy as there was no indication either preoperatively or intraoperatively for the same. The anatomy consisted of a single right hepatic artery (3 mm), single right portal vein (8 mm; Type A), and 2 spectacled ducts (right anterior and posterior hepatic ducts close to each other—2 mm each; Type A3). The right hepatic vein was 8 mm in diameter. Both segment 5 and segment 8 veins were reconstructed as a neo-middle hepatic vein on the backtable using a polytetrafluoroethylene (PTFE) graft. The actual graft weight was 687 g, with a graft-to-recipient weight ratio (GRWR) of 1.09. We flushed the graft with 2000 ml of University of Wisconsin solution on the backtable.

This was followed by implantation on the recipient in standard fashion. The portal vein eversion thrombectomy had already been performed. The cold ischemia time was 138 mins, and the warm ischemia time was 48 mins. The anhepatic phase duration was 110 mins. The reperfusion was uniform and immediate. After completing arterial and biliary reconstructions, ultrasound (USG) Doppler showed excellent flows and the graft was pink and soft on inspection (Figure 3(b)).

Postoperative course was uneventful for the donor. She was extubated on the table and had no complications. Lactate normalized on postoperative day (POD) 2 and international normalized ratio (INR) was normal on POD-1. Liver function tests (LFT) gradually improved and the diet was started on POD-2. Gradually, she improved and was mobilized. She was discharged on POD-6 in stable condition. Her postoperative labs are summarized in Table 2 (Table 2). The recipient was kept intubated on the day of surgery and needed re-exploration on POD-1 for surgical bleeding. Lactate, LFT, and INR continued to improve and apart from the short duration of acute kidney injury (AKI) which resolved with judicious fluid management, he had no postoperative issues (Table 3). He was eventually discharged on POD-15.

## 3. Discussion

Significant hepatic macrosteatosis is a known contra-indication to living donor hepatectomy [2, 4]. Most studies are retrospective analyses which have compared outcomes of the degree of hepatic steatosis to the functional outcome in both the recipient as well as the donor. The incidence of wound-related complications has also been reported to be higher in donors with a BMI greater than 30 kg/m^2^ [2]. Rela et al. suggested that the criteria acceptable for evaluation may be relaxed to 35 kg/m^2^ in case the donor is very muscular or there are no other suitable donors [4]. Almost all centers reject donors with a BMI greater than 35 kg/m^2^ [1, 4]. The donor screening for hepatic steatosis is usually performed by USG, CT scan (LAI or L/S ratio), and MR spectroscopy [1]. USG is rarely used due to its subjective nature and is only useful as a screening tool. The CT scan is the most commonly preferred modality, and LAI is the most widely used tool for the assessment of liver macrosteatosis [1]. MRI is the most accurate among the imaging modalities but also more expensive. It can also assess for microsteatosis and, hence, give a better idea about the overall quality of the graft [7, 8]. Higher volume centers usually prefer MRI more than low-volume centers [1]. Donors with a higher BMI are usually advised a combined regimen of diet and exercise for weight loss [5, 9]. There are also medications to aid in weight loss for highly motivated donors [5, 9].

In our patient, the recipient was getting sicker with no other suitable donor, so we proceeded to evaluate her even with a BMI of 36.5 kg/m^2^. With an LAI of +16 and a liver remnant of 37%, we proceeded to confirm our fat estimation by MR spectroscopy. When fat fraction evaluation was reported as 3% (mean) in both lobes and the volumetric analysis was acceptable, we agreed to accept the donor as she did not have any other comorbidity. We further revisited the CT scan to assess the V/S ratio. The CT showed a high amount of subcutaneous fat area (546 cm^2^) compared to the visceral fat area (202 cm^2^) at the level of *L*4. This gave us a V/S ratio of 0.37, which was lower than 0.4 (applicable only to obese individuals) indicating that our potential donor had subcutaneous fat obesity and not visceral fat obesity [6, 10, 11]. Higher visceral fat is positively correlated with higher fasting blood glucose levels, triglyceride levels, and cholesterol levels [11, 12].

The correlation between hepatic steatosis and obesity is well known. However, many studies have focused on the fat distribution and their relationship to liver fat estimates. Recent literature suggests that visceral obesity and V/S ratio have a direct positive correlation to hepatic fat content, while subcutaneous fat alone does not significantly impact it [13–17]. There is a slightly higher risk of wound-related complications (infection or hernia) in obese patients, but our donor did not have any such issues so far [18–20].

Our donor had a normal postoperative course without any complications. She was mobilized on POD-2, drain removed on POD-4, and discharged on POD-6. At 3 weeks of follow-up, her wound is clean and well-healed, and she is already back to doing her daily life activities without any pain. Her follow-up investigations are normal (See Table 2). The recipient liver function was also immediate and his postop course was within expected lines. Posttransplant, his elbow has improved significantly with no further fluid or pus collections or pain. His postoperative liver Dopplers were normal and he was discharged on POD-15. His LFT has almost normalized at 3 weeks postsurgery. To the best of our knowledge, this is one of the first reports of a living liver donation from a donor with a BMI of more than 35 kg/m^2^.

## 4. Conclusion

In conclusion, subcutaneous fat obesity should not be considered as a contraindication to liver donation even with a BMI >35 kg/m^2^. When no other suitable donors are available, it may be worthwhile to evaluate potentials donors even with a higher BMI to assess for hepatic steatosis. A small percentage of healthy individuals will not have visceral fat obesity and may not have steatotic livers. The CT scan and MR fat fraction estimation can confirm the findings. A biopsy may be avoided if MR fat estimation is <10% in obese donors. Intraoperative visualization in these donors remains the gold standard to decide the need for biopsy. Living donor hepatectomy may be safely performed in a select group of high-BMI patients (>35 kg/m^2^) with pure subcutaneous fat obesity in the absence of other suitable living donors.

## Figures and Tables

**Table 1:** Volumetric analysis of donor.

Part of liver	Volume (cc)	Percentage	GRWR (wt 63 kg)
Total liver	1332.5	100	—
Caudate	47.8	3.6	—
Right lobe without MHV	779.2	58.5	1.23
Left lobe with MHV	505.5	37.9	0.80

(GRWR: graft-to-recipient weight ratio; MHV: middle hepatic vein).

**Table 2:** Donor preoperative and postoperative investigations.

Test name	Preop	POD-1	POD-3	POD-6 (disch)	3 weeks (*F*/up)
Lactate	—	2.0	0.9	—	—
Total bilirubin	0.3	1.4	2.0	0.5	0.4
SGOT	14	211	130	41	31
SGPT	13	162	128	73	34
INR	0.90	1.03	—	—	0.84
Creat	0.77	0.58	0.68	0.65	0.78

*POD: postoperative day; disch: discharge; F/up: follow-up.*

**Table 3:** Recipient preoperative and postoperative investigations. He had acute kidney injury on postoperative 3 which resolved with judicious fluid management.

Test name	Preop	POD-1	POD-2	POD-3	POD-5	POD-10	POD-15 (disch)	POD-22 (*F*/up)
Lactate	1.1	2.6	1.7	1.1	—	—	—	—
Total bilirubin	1.2	5.7	5.8	10.5	9.4	7.1	5.0	3.0
SGOT	42	128	110	68	60	67	83	30
SGPT	18	93	132	105	78	58	75	47
INR	1.0	2.73	2.31	1.66	1.24	—	—	—
Creat	1.48	0.92	1.73	1.79	1.15	1.01	0.67	0.69

POD: postoperative day; disch: discharge; *F*/up: follow-up.

## Data Availability

The data supporting the findings of this study are available within the article. Any supplementary data that may support the finding of this study may be available from the corresponding author upon reasonable request.
